# An Efficient and Secure Semi-Quantum Secret Sharing Scheme Based on *W* State Sharing of Specific Bits

**DOI:** 10.3390/e27111107

**Published:** 2025-10-26

**Authors:** Kai Xing, Rongbo Lu, Sihai Liu, Lu Lan

**Affiliations:** 1 School of Communication and Electronic Engineering, Jishou University, Jishou 416000, China; 2 School of Computer Science and Artificial Intelligence, Huaihua University, Huaihua 418099, China

**Keywords:** semi-quantum secret sharing, three-particle *W* states, decoy photon, double controlled-NOT attacks

## Abstract

This paper presents a semi-quantum secret sharing (SQSS) protocol based on three-particle *W* states, designed for efficient and secure secret sharing in quantum-resource-constrained scenarios. In the protocol, a fully quantum-capable sender encodes binary secrets using *W*, while receivers with limited quantum capabilities reconstruct the secret through collaborative *Z* basis measurements and classical communication, ensuring no single participant can obtain the complete information independently. The protocol employs a four-state decoy photon technique ({|0〉,|1〉,|+〉,|−〉}) and position randomization, combined with photon number splitting (PNS) and wavelength filtering (WF) technologies, to resist intercept–resend, entanglement–measurement, and double controlled-NOT(CNOT) attacks. Theoretical analysis shows that the detection probability of intercept–resend attacks increases exponentially with the number of decoy photons (approaching 1). For entanglement–measurement attacks, any illegal operation by an attacker introduces detectable quantum state disturbances. Double CNOT attacks are rendered ineffective by the untraceability of particle positions and mixed-basis strategies. Leveraging the robust entanglement of *W* states, the protocol proves that the mutual information between secret bits and single-participant measurement results is strictly zero, ensuring lossless reconstruction only through authorized collaboration.

## 1. Introduction

In the current digital landscape, safeguarding personal privacy, corporate data assets, and national security infrastructures has become paramount. The rapid advancement of information technology has exposed traditional cryptographic schemes to escalating challenges, particularly with the emergence of quantum computing, which poses a fundamental threat to classical encryption algorithms. To counter these threats, researchers have turned to novel cryptographic paradigms. Among these, secret sharing [1] has emerged as a cornerstone of distributed cryptography, offering robust solutions for secure multi-party collaboration and privacy preservation. By partitioning secret information among multiple participants, this technique ensures that only authorized subsets of participants can reconstruct the original secret. However, the growing capabilities of quantum computers have undermined the security of classical secret sharing protocols. To address this issue, Hillery et al. [2] introduced the first quantum secret sharing (QSS) protocol in 1999, employing Greenberger–Horne–Zeilinger (GHZ) states. This pioneering work has greatly accelerated the development of subsequent theoretical work in this field [3,4,5].

Meanwhile, experimental efforts have progressively translated these theoretical constructs into practical implementations. In 2018, Zhou et al. [6] leveraged the multipartite bound entanglement of optical fields to share quantum secrets among four participants, providing a convenient and advantageous pathway for practical QSS applications. The following year, Williams et al. [7] experimentally realized QSS using polarization-entangled photon pairs, demonstrating the essential correlations for three-party secret sharing while highlighting the protocol’s straightforward extensibility to additional parties. More recently, in 2024, Qin et al. [8] proposed an efficient, secure, and flexible QSS scheme involving eight users, facilitated by a continuous-variable eight-partite bound entangled state. Building on these advances, Xiao et al. [9] in 2025 presented an experimental demonstration where their protocol’s remarkable scalability advantages were particularly evident within a fully connected quantum network, enabling real-time and efficient QSS among multiple users.

However, existing QSS protocols mandate that all participants possess full quantum capabilities—including quantum state generation, manipulation, and measurement—requirements that remain prohibitively expensive and challenging to scale with current technology. To reconcile cryptographic security with practical quantum resource constraints, Boyer et al. [10] pioneered semi-quantum key distribution (SQKD) in 2007. This framework establishes a fundamental distinction between participant capabilities: while one party maintains full quantum capabilities, other participants (termed “classical” or “semi-quantum”) are restricted to specific, minimal quantum operations. The rationale for restricting semi-quantum participants to SIFT (measure and resend in the *Z* basis) and CTRL (reflect) operations is twofold: theoretically, it explores the minimal quantum resources required for secure protocols; practically, it models realistic deployment scenarios where participants may have access to basic quantum measurement devices but lack sophisticated quantum preparation capabilities. Crucially, these restricted operations are physically realizable with current technology: SIFT can be implemented with a single-photon detector and a classical pulse source, while CTRL can be trivially achieved using passive optical components like mirrors or optical switches. This paradigm has catalyzed subsequent SQKD protocol developments [11,12], with rigorous unconditional security proofs established in subsequent works [13,14]. Here, “unconditional security” signifies that the protocol’s security is guaranteed by the laws of quantum mechanics, not by computational assumptions, making it resilient even against attackers with unlimited computational power. This robust security stems from the fact that any eavesdropping attempt inevitably disturbs the transmitted quantum states, a disturbance that is detected through classical post-processing (e.g., eavesdropping detection and privacy amplification). Crucially, the introduction of semi-quantum participants enhances practicality without compromising this information-theoretic security foundation.

In 2010, Li et al. [15] pioneered the first semi-quantum secret sharing (SQSS) protocol utilizing GHZ states, thereby relaxing quantum capability requirements. Subsequent analysis by Lin et al. [16] identified security flaws in [15] and proposed countermeasures. To reduce implementation complexity, Li et al. [17] developed an SQSS protocol using single-particle product states. Xie et al. [18] introduced a three-particle entangled state SQSS protocol for sharing a specific secret message. Yin et al. [19] demonstrated vulnerabilities in [18] against adversarial participants and proposed an GHZ-enhanced variant. Yin et al. [20] advanced SQSS efficiency via Bell-state implementations. Tsai et al. [21] designed the first *W*-state SQSS protocol, sacrificing efficiency for stronger entanglement. Tian et al. [22] formulated a flexible Bell-state SQSS scheme, later shown by He et al. [23] to be vulnerable to double controlled-NOT (CNOT) attacks. Li et al. [24] constructed a cluster-state SQSS protocol with quantum entanglement swapping technology, though it faced resource-intensive challenges. Chen et al. [25] proposed a χ-state SQSS framework. Recent advances include Hou et al. [26]’s hybrid GHZ-based circular SQSS and Ma et al. [27]’s third-party-assisted protocol for participants with limited quantum capabilities.

This paper proposes an efficient and secure SQSS protocol based on *W* states, representing a significant extension of the semi-quantum paradigm to multi-party scenarios. Unlike existing SQSS protocols that predominantly focus on two-party scenarios or require complex quantum resources, our protocol leverages the robust entanglement properties of *W* states while maintaining minimal quantum requirements for two of the three participants. Specifically, only Alice requires full quantum capabilities for state preparation, while Bob and Charlie operate as semi-quantum participants restricted to SIFT and CTRL operations. This configuration substantially reduces the practical implementation barriers compared to fully quantum protocols. For security verification, we employ decoy photon integration, rigorously demonstrating resilience against conventional attack vectors including double CNOT attacks. Benchmarked against existing SQSS schemes, our protocol demonstrates both enhanced operational efficiency and practical viability while maintaining strong security guarantees.

The paper proceeds as follows. Section 2 presents the foundational theoretical frameworks essential to the proposed scheme. Section 3 details the design and operational steps of our SQSS protocol. Section 4 provides a rigorous security analysis, evaluating the protocol’s resilience against potential attacks. Section 5 explores the protocol’s efficiency through quantitative metrics and conducts a comparative analysis with existing schemes. Finally, Section 6 concludes by synthesizing the findings and discussing future research directions.

## 2. Preliminary Theory

### 2.1. Quantum Gates

Quantum logic gates constitute the foundational operational units in quantum computation, enabling state manipulation and evolution. This section delineates four critical quantum gates: the *Y*-axis rotation gate ($R_{y}$), Pauli-*X* (*X*) gate, CNOT gate, and controlled-*Z* (CZ) gate.

The $R_{y}$ gate performs a unitary transformation on a single qubit by rotating its Bloch sphere representation about the *Y*-axis by angle θ. Its matrix representation is

(1) $$R_{y}\left(\theta \right)=\left(\begin{array}{cc} \cos\left(\frac{\theta }{2}\right) & -\sin\left(\frac{\theta }{2}\right)\\ \sin\left(\frac{\theta }{2}\right) & \cos\left(\frac{\theta }{2}\right) \end{array}\right).$$

The *X* gate inverts the computational basis states |0〉 and |1〉, defined by

(2) $$X=\left(\begin{array}{cc} 0 & 1\\ 1 & 0 \end{array}\right).$$

The CNOT gate operates on two qubits, where the state of the target qubit undergoes an *X* transformation conditioned on the control qubit being |1〉. Its matrix form is

(3) $$\mathrm{CNOT}=\left(\begin{array}{cccc} 1 & 0 & 0 & 0\\ 0 & 1 & 0 & 0\\ 0 & 0 & 0 & 1\\ 0 & 0 & 1 & 0 \end{array}\right).$$

The CZ gate introduces a conditional phase shift, applying a *Z* gate to the target qubit only when the control qubit is |1〉. Its matrix representation is

(4) $$\mathrm{CZ}=\left(\begin{array}{cccc} 1 & 0 & 0 & 0\\ 0 & 1 & 0 & 0\\ 0 & 0 & 1 & 0\\ 0 & 0 & 0 & -1 \end{array}\right).$$

To construct the *W* state, a specialized two-qubit gate (F gate) is designed by combining rotations and the CZ gate. This gate enables controlled probability distribution and entanglement between qubits, critical for generating the equal-amplitude superposition of single-excitation states in *W* states. It can be expressed as

(5) $$F_{i,j}\left(\theta \right)=\left(I_{i}⊗R_{y}^{\left(j\right)}\left(\theta \right)\right)CZ_{i,j}\left(I_{i}⊗R_{y}^{\left(j\right)}\left(-\theta \right)\right)$$

*I* denotes the unit matrix (which does not change the qubit state it acts on); ⊗ represents the tensor product; and i,j∈{0,1,2,…} are the labels of the two distinct qubits targeted by the F gate.

The selection of angle θ is the core for achieving equal probability distribution in the F gate, and its analytical expression is

(6) $$\theta =\arccos\left(\sqrt{\frac{1}{n-k+1}}\right)$$

Here, *n* represents the length of the particle, and *k* indicates the number of times the F gate is used.

These quantum gates form the building blocks for constructing the *W* states utilized in our protocol (see Section 2.2). Specifically, the CNOT and CZ gates are critical for entangling particles during *W* state preparation, while the *X* gate enables state transformations between $|W_{0}\rangle$ and $|W_{1}\rangle$ for secret encoding (see Equations (7) and (8)). Their combined operations ensure the robust entanglement and specific bit encoding necessary for secure SQSS, as detailed in Section 3.

### 2.2. W State

The proposed protocol employs the three-particle *W* state [28] as the specific quantum resource for information encoding. We define $|W_{1}\rangle$ as the three-particle *W* state, given by

(7) $$|W_{1}\rangle =\frac{1}{\sqrt{3}}\left(|001\rangle +|010\rangle +|100\rangle \right)\;$$

**Figure 1:**
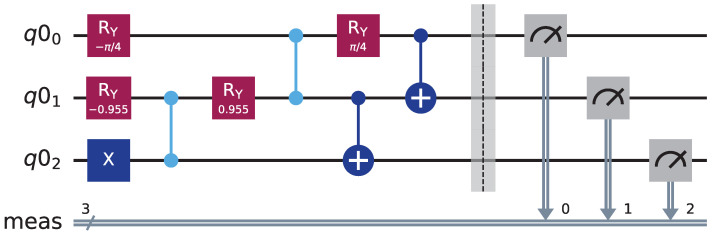
Circuit for preparation the *W* state.

Figure 1 illustrates the quantum circuit for *W*-state preparation, constructed through systematic gate operations on three initialized qubits ($q0_{0}$, $q0_{1}$, $q0_{2}$) in the state |0〉|0〉|0〉. First, apply an *X* gate to $q0_{2}$. Second, apply the F gate to the qubit pairs ($q0_{2}$, $q0_{1}$) and ($q0_{1}$, $q0_{0}$). Then, apply the CNOT gate to the qubit pairs ($q0_{1}$, $q0_{2}$) and ($q0_{0}$, $q0_{1}$), respectively. Finally, the *W* state is obtained at the output of the circuit.

**Figure 2:**
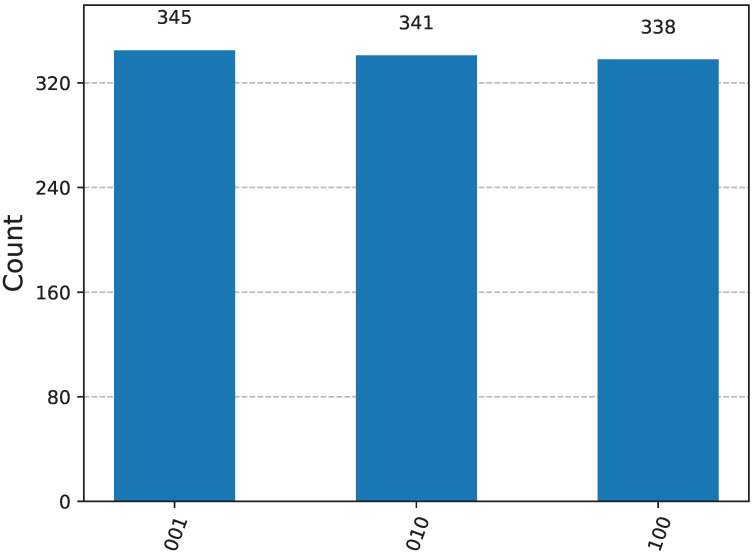
Probability distribution of *W*-state measurement results from a 1024-Sample IBM Qiskit Simulation.

To verify the correctness of the quantum circuit, we conducted 1024 simulations using IBM Qiskit (Version: 2.1.2) on PyCharm. The test results are shown in Figure 2. The three measurement outcomes of the *W* state, labeled as |001〉, |010〉, |100〉, exhibit approximately equal probabilities, demonstrating that the circuit can effectively prepare the *W* state.

An alternative *W*-state variant serves as auxiliary quantum resource. The state $|W_{0}\rangle$ is obtained by applying an *X* gate to the third qubit of $|W_{1}\rangle$ as follows:

(8) $$|W_{0}\rangle =I⊗I⊗X|W_{1}\rangle =\frac{1}{\sqrt{3}}\left(|000\rangle +|011\rangle +|101\rangle \right)\;$$

### 2.3. Semi-Quantum Model

**Figure 3:**
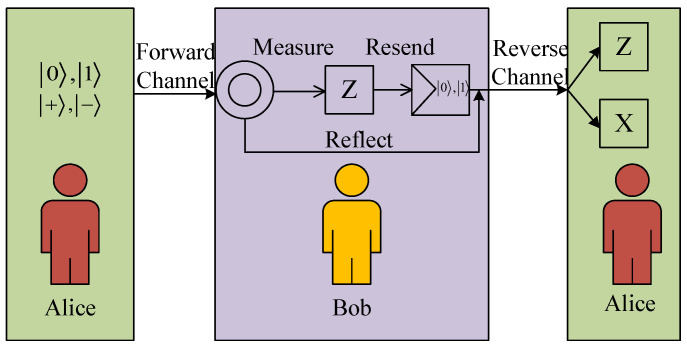
Semi-quantum communication protocol schematic. Alice creates a single-photon quantum state and transmits it to Bob over the forward channel. In this process, SIFT corresponds to the measure-and-resend mode: Bob measures the qubit in the *Z* basis, reconstructs the quantum state according to the measurement outcome, and then sends it back to Alice via the backward channel. CTRL, on the other hand, is the reflection mode: Bob takes no action and directly reflects the qubit to Alice through the backward channel. Upon receiving the qubit from the backward channel, Alice randomly chooses a measurement basis to perform the measurement.

This semi-quantum communication model [10] is illustrated in Figure 3. Within this architecture, Alice maintains full quantum operational capacity for executing arbitrary quantum transformations, while Bob operates under quantum constraints limited to primitive operations.

## 3. Protocol

In the proposed protocol, three participants are included: Alice is equipped with full quantum capabilities, enabling the execution of complex quantum operations, while Bob and Charlie have restricted quantum capabilities and can only perform simple quantum operations. Specifically, in this study, Bob and Charlie acquire secret information by measuring the entangled states prepared by Alice. Each party can only access partial information, and the complete secret can only be reconstructed through collaboration between them; neither party can independently obtain the secret.

**Figure 4:**
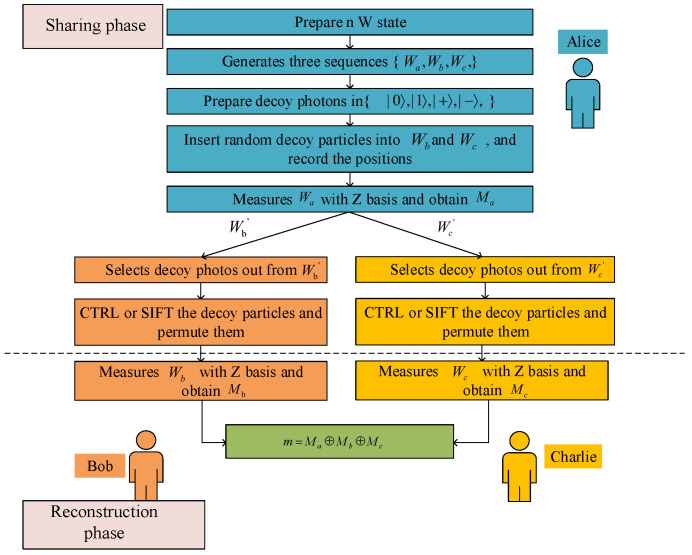
The process of the proposed SQSS protocol.

Moreover, the proposed scheme is not limited to ideal quantum channels. Owing to the incorporation of decoy photon sequences, photon number splitting (PNS) and wavelength filtering (WF) techniques, as well as classical error correction and privacy amplification mechanisms, the protocol remains feasible and secure even under imperfect communication channels affected by noise, decoherence, or photon loss. The intrinsic correlation of the three-particle *W* states ensures that the correctness and security of the reconstructed secret are maintained as long as the channel fidelity exceeds a reasonable threshold. The steps of the proposed SQSS protocol are detailed in Figure 4, with the specific procedures elaborated as follows.

### 3.1. Sharing Phase

Step 1. Alice generates the secret message sequences $m=\left\{m_{1},m_{2},\ldots ,m_{n}\right\}\in {\left\{0,1\right\}}^{n}$ and prepares the corresponding *W* states, where $|W_{0}\rangle$ is generated for 0 and $|W_{1}\rangle$ is generated for 1, resulting in a sequence $W={\{|W\rangle }_{1}{,|W\rangle }_{2}{,\ldots ,|W\rangle }_{n}\}\in \{|W_{0}\rangle \;|W_{1}{\rangle \}}^{n}$. She then divides *W* into three sequences $W_{a}={\{|W\rangle }_{1}^{a}{,|W\rangle }_{2}^{a}{,\ldots ,|W\rangle }_{n}^{a}\}$, $W_{b}={\{|W\rangle }_{1}^{b}{,|W\rangle }_{2}^{b}{,\ldots ,|W\rangle }_{n}^{b}\}$, and $W_{c}={\{|W\rangle }_{1}^{c}{,|W\rangle }_{2}^{c}{,\ldots ,|W\rangle }_{n}^{c}\}$, where $W_{a}$, $W_{b}$, and $W_{c}$ are constructed from the first, second, and third particles of each *W* state in sequence *W*, respectively.

Step 2. Alice prepares two decoy photon sequences of length *l*, where each decoy photon is randomly selected from the four single-photon states {|0〉,|1〉,|+〉,|−〉}. She then randomly inserts the decoy photon sequences into the $W_{b}$ and $W_{c}$ sequences, generating new sequences $W_{b}^{\prime }$ and $W_{c}^{\prime }$. The positions and states of the decoy photons are recorded for later verification of channel security.

Step 3. To defend against Trojan horse attacks [29,30,31,32], Alice’s setup is equipped with a PNS and WF. Alice sends $W_{b}^{\prime }$ and $W_{c}^{\prime }$ to Bob and Charlie, respectively. After Bob and Charlie receive their respective sequences, they announce the completion of the reception.

Step 4. Alice announces the positions of the decoy photons to Bob and Charlie. Bob and Charlie identify their respective decoy particles, perform a random permutation, and record the new positions. They then apply either the CTRL or SIFT operation to these particles. After completing the operations, they send the particles back to Alice through the reverse channel.

Step 5. Upon receiving the decoy particles, Bob and Charlie inform Alice of the new positions of their respective permuted sequences and the operations (CTRL or SIFT) they performed. Alice subsequently conducts measurements on these decoy particles using basis sets corresponding to the declared operations, then cross-verifies the obtained results against her initially prepared states, as specified in Table 1. If the error rate exceeds a preestablished threshold, the protocol is terminated immediately. Conversely, if the error rate remains within acceptable limits, the quantum channel is deemed safe, and the protocol proceeds to the subsequent phase.

### 3.2. Reconstruction Phase

Step 6. After passing the eavesdropping check, Alice performs a *Z* basis measurement on her sequence $W_{a}$, records the measurement results as $M_{a}$, and publicly announces them. When Bob and Charlie collaborate to recover Alice’s secret, they perform *Z* basis measurements on their respective particle sequences $W_{b}$ and $W_{c}$. Using error correction codes and privacy amplification techniques [33,34], they record their measurement results as $M_{b}$ and $M_{c}$.

Step 7. To verify the validity of the shared secret, Bob and Charlie select a subset of test bits from $M_{b}$ and $M_{c}$, combine them with $M_{a}$, and perform XOR operations on these bits. They then check if the resulting values match the corresponding bits of *m*. If they match, they proceed to recover the secret sequence by computing $m=M_{a}⊕M_{b}⊕M_{c}$, as illustrated in Table 2.

### 3.3. An Example

Here, we provide an example to illustrate our scheme. Suppose Alice’s secret information is m={0,1,1,0,0,0}. Based on *m*, she prepares the sequence $W=\{W_{0},W_{1},W_{1},W_{0},W_{0},W_{0}\}$ and divides it into three sequences $W_{a}$, $W_{b}$ and $W_{c}$. Alice then prepares two decoy sequences of length 4 and randomly inserts them into $W_{b}=\left\{W_{0}^{b},W_{1}^{b},W_{1}^{b},W_{0}^{b},W_{0}^{b},W_{0}^{b},\right\}$ and $W_{c}=\left\{W_{0}^{c},W_{1}^{c},W_{1}^{c},W_{0}^{c},W_{0}^{c},W_{0}^{c}\right\}$, generating new sequences $W_{b}^{\prime }=\{W_{0}^{b},W_{1}^{b},|+\rangle ,W_{1}^{b},|0\rangle ,|1\rangle ,W_{0}^{b},W_{0}^{b},W_{0}^{b},|-\rangle \}$ and $W_{c}^{\prime }=\{W_{0}^{c},|-\rangle ,W_{1}^{c},W_{1}^{c},|0\rangle ,W_{0}^{c},|1\rangle ,W_{0}^{c},W_{0}^{c},|+\rangle \}$, while recording the positions of the decoy photon. Alice sends these two sequences to Bob and Charlie, respectively. After passing the eavesdropping check, Alice measures her sequence and publicly announces $M_{a}=\left\{0,1,0,0,0,1\right\}$. When Bob and Charlie want to recover Alice’s secret, they perform *Z* basis measurements on their respective particle sequences, obtaining measurement results $M_{b}=\left\{0,0,0,1,1,1\right\}$ and $M_{c}=\left\{0,0,1,1,1,0\right\}$. Thus, the secret information *m* is uniquely retrieved through the collaborative computation $m=M_{a}⊕M_{b}⊕M_{c}$, leveraging the pre-defined XOR reconstruction rule among the three participants’ measurement results.

## 4. Security Analysis

This section evaluates the protocol’s security against two types of attackers: external adversaries and internal attackers. Given internal attackers’ privileged access to protocol information, we focus on internal attacks. Consider a scenario where a dishonest participant Bob operates as an internal attacker with full quantum capabilities. Below, we describe the security analysis of the proposed protocol against such attacks.

### 4.1. Intercept–Resend Attack

In this type of attack, the internal attacker Bob intercepts the qubit sequence $W_{c}^{\prime }$ transmitted from Alice to Charlie. He performs basis measurements to extract secret information, then resends measured states to Charlie. Such interventions inevitably perturb decoy photon states, enabling detection through two observable scenarios Table 3.

Consider the scenario where Alice prepares the initial state of the decoy photon as |0〉 (or |1〉). Bob has a probability of 50% of choosing to measure in the same basis as the decoy state. In such a case, regardless of the operation Charlie selects, the eavesdropping attempt by Bob will remain undetected. On the other hand, Bob also has a probability of 50% of choosing a measurement basis different from that of the decoy state. In this situation, regardless of whether Charlie opts for the CTRL or SIFT operation, there is a probability of 50% of introducing a disturbance to the state. Consequently, the probability $p_{0}$ of being detected is calculated as follows: $p_{0}=\frac{1}{2}\times \frac{1}{2}=\frac{1}{4}.$

Now, assume that Alice prepares the initial state of the decoy photon as |+〉(or |−〉). Bob has a 50% probability of choosing to measure in the same basis as the decoy state. In this instance, regardless of the operation Charlie performs, the eavesdropping attack will not be detected. Additionally, Bob has a 50% probability of choosing to measure in a basis different from the decoy state. If Charlie chooses the CTRL operation, there is a probability of 50% of causing a disturbance; if Charlie chooses the SIFT operation, the eavesdropping attack will not be detected. Therefore, the probability $p_{1}$ of being detected is calculated as follows: $p_{1}=\frac{1}{2}\times \frac{1}{2}\times \frac{1}{2}=\frac{1}{8}.$

It should be noted that the length of the decoy particle sequence is *l*. Assuming that the decoy particles in the *Z* basis and those in the *X* basis each account for half of the total decoy particles, based on the analysis, we can infer that the probability *p* of detecting Bob’s attack is

(9) $$\;p=1-{\left(1-p_{0}\right)}^{\frac{l}{2}}{\left(1-p_{1}\right)}^{\frac{l}{2}}=1-{\left(\frac{3}{4}\right)}^{\frac{l}{2}}{\left(\frac{7}{8}\right)}^{\frac{l}{2}}=1\left(l\to \infty \right)\;$$

This demonstrates that the protocol’s interception detection probability approaches certainty as the number of decoy photons *l* increases.

### 4.2. Entangle–Measure Attack

Suppose Bob, a malicious participant, attempts to illicitly extract secret information through entanglement–measurement attacks [10,22,23,26,27] on the quantum channels between Alice and Charlie. Specifically, he prepares ancillary particles, entangles them with the transmitted states, and extracts information via measurements on these auxiliary systems.

**Figure 5:**
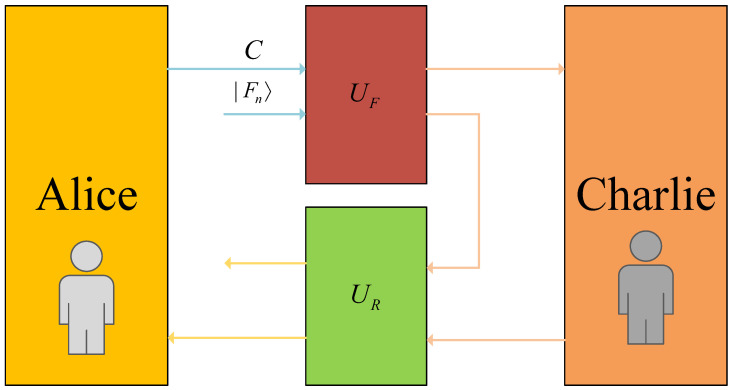
Bob’s entangle–measure attack.

In the security analysis, Bob’s potential eavesdropping on the forward and reverse channels is mathematically formulated as unitary transformations $U_{F}$ and $U_{R}$, corresponding to the forward and reverse directions, respectively. Given the protocol’s exclusive reliance on decoy photons for eavesdropping detection, the analysis focuses on how entanglement–measurement attacks impact the states of these decoy photons. This attack mechanism is illustrated in Figure 5.

When the decoy particles are transmitted to Charlie, Bob performs the $U_{F}$ operation on the decoy particles *C* and the detection particles *F*, and the system evolves as follows:

(10) $$\;U_{F}{|0\rangle }_{C}|F_{n}\rangle =|0\rangle |F_{00}\rangle +|1\rangle |F_{01}\rangle \;$$

(11) $$\;U_{F}{|1\rangle }_{C}|F_{n}\rangle =|0\rangle |F_{10}\rangle +|1\rangle |F_{11}\rangle \;$$

(12) $$\;U_{F}{|+\rangle }_{C}|F_{n}\rangle =|0\rangle |F_{00}\rangle +|1\rangle |F_{01}\rangle +|0\rangle |F_{10}\rangle +|1\rangle |F_{11}\rangle \;$$

(13) $$\;U_{F}{|-\rangle }_{C}|F_{n}\rangle =|0\rangle |F_{00}\rangle +|1\rangle |F_{01}\rangle -|0\rangle |F_{10}\rangle -|1\rangle |F_{11}\rangle \;$$

Here, $|F_{ij}\rangle$ denotes the state of Bob’s auxiliary particles. Upon Charlie’s reception of the qubits transmitted by Alice, he may apply either the SIFT or CTRL operation to the particles at hand. Should Charlie select the SIFT operation, the system will undergo a transformation to

(14) $$\;U_{R}\left|a\rangle \right|F_{ab}\rangle =|a\rangle |R_{ab}\rangle ,a,b\in \left\{0,1\right\}\;$$

The critical requirement for the attack operation $U_{R}$ is that it must leave the state of system *C* unchanged. When Charlie chooses the CTRL operation, the composite system evolves as

(15) $$\;U_{R}\left|0\rangle \right|F_{n}\rangle =|0\rangle |R_{00}\rangle +|1\rangle |R_{01}\rangle \;$$

(16) $$\;U_{R}\left|1\rangle \right|F_{n}\rangle =|0\rangle |R_{10}\rangle +|1\rangle |R_{11}\rangle \;$$

(17) $$\;U_{R}\left|+\rangle \right|F_{n}\rangle =\frac{1}{2}[|+\rangle (|R_{00}\rangle +|R_{01}\rangle +|R_{10}\rangle +|R_{11}\rangle )+|-\rangle (|R_{00}\rangle -|R_{01}\rangle +|R_{10}\rangle -|R_{11}\rangle )]\;$$

(18) $$\;U_{R}\left|-\rangle \right|F_{n}\rangle =\frac{1}{2}[|+\rangle (|R_{00}\rangle +|R_{01}\rangle -|R_{10}\rangle -|R_{11}\rangle )+|-\rangle (|R_{00}\rangle -|R_{01}\rangle -|R_{10}\rangle +|R_{11}\rangle )]\;$$

If Bob wants to succeed in the eavesdropping detection, then Equations (15)–(18) should be satisfied:

(19) $$\;|R_{01}\rangle =|R_{10}\rangle =|R_{00}\rangle -|R_{01}\rangle +|R_{10}\rangle -|R_{11}\rangle =|R_{00}\rangle +|R_{01}\rangle -|R_{10}\rangle -|R_{11}\rangle =0\;$$

According to Equation (19), it can be deduced that

(20) $$\;|R_{00}\rangle =|R_{11}\rangle =|\tau \rangle \;$$

By substituting Equation (20) into Equations (15)–(18), they can be written as

(21) $$\;U_{R}\left|0\rangle \right|F_{n}\rangle =|0\rangle |\tau \rangle \;$$

(22) $$\;U_{R}\left|1\rangle \right|F_{n}\rangle =|1\rangle |\tau \rangle \;$$

(23) $$\;U_{R}\left|+\rangle \right|F_{n}\rangle =|+\rangle |\tau \rangle \;$$

(24) $$\;U_{R}\left|-\rangle \right|F_{n}\rangle =|-\rangle |\tau \rangle \;$$

Equations (21)–(24) results demonstrate that Bob’s auxiliary particles must always reside in the state |τ〉 to avoid detection, implying that the final state of his detection system is entirely independent of Charlie’s measurement outcomes. Therefore, the protocol maintains resilience against entanglement–measure attacks by ensuring that any such malicious operations leave no detectable trace on the decoy photon states.

However, the aforementioned analysis only considers the ideal scenario where Bob attempts to avoid detection completely. In practice, Bob may employ more general individual attack strategies, accepting a certain error rate to gain partial information. To quantitatively analyze such attacks, we adopt the framework established by Slutsky et al. [35], which defines a rigorous relationship between the error rate and the maximum information obtainable by an eavesdropper.

In Slutsky’s model, the eavesdropper performs independent operations on each qubit, entangling it with a probe system through a unitary evolution *U*. Due to protocol symmetry, the most general attack can be parameterized using four parameters (λ,μ,θ,ϕ). These parameters define the coefficients of the probe system states (see Equation (4) in Ref. [35]) and fully determine both the attack’s impact on quantum states and the induced error rate.

Although these parameters are essential for optimizing attack strategies, Slutsky et al. have demonstrated through optimization methods that for any given error rate E, there exists a definite upper bound $I_{max}^{R}\left(E\right)$ on the maximum Renyi information obtainable about the final key. Therefore, in our security proof, we can directly utilize the established relationship $I_{max}^{R}\left(E\right)$ without explicitly handling these parameters.

In our protocol, the decoy photons are prepared in four non-orthogonal states {|0〉,|1〉,|+〉,|−〉}. Thus, Slutsky’s analysis is directly applicable. Let $E_{f}$ and $E_{b}$ represent the error rates of the forward and backward channels, respectively. Alice and Charlie can independently estimate these error rates by comparing the decoy photons transmitted in both directions.

According to their findings, for a given error rate *E*, the upper bound on the maximum Renyi information $I_{R}^{\max}\left(E\right)$ satisfies

(25) $$\;I_{max}^{R}\left(E\right)=\log_{2}\left(1+cos^{2}2\xi \right),sin2\xi =Q_{min}\left(E\right)\;$$

where $Q_{min}\left(E\right)$ denotes the minimum state overlap achieved through parameter optimization. For the bidirectional channel, Bob’s information gain from forward and backward attacks is cumulative in the worst case. Thus, the total information upper bound becomes

(26) $$\;I_{total}^{R}\left(E\right)\leq I_{max}^{R}\left(E_{f}\right)+I_{max}^{R}\left(E_{b}\right)\;$$

During privacy amplification, Alice and Charlie determine the compression ratio based on the observed total error rate $E_{\mathrm{total}}=E_{f}+E_{b}$ and the corresponding information bound $I_{total}^{R}$. Specifically, they compress the raw key to approximately $1-I_{total}^{R}$ times its original length. This ensures that even under optimal individual attacks on both channels, Bob’s information about the final key remains exponentially suppressed.

Consequently, our protocol provides unconditional security against all individual attacks—not only resisting idealized entanglement–measurement attacks with zero errors but also leveraging actual error rates and privacy amplification to maintain security under practical conditions.

### 4.3. Double CNOT Attack

First proposed by Boyer et al. [10], the double CNOT attack represents a sophisticated eavesdropping approach designed to exploit vulnerabilities in semi-quantum communication frameworks. By deploying cascaded CNOT operations, malicious actors can disrupt quantum signal transmission, aiming to extract information surreptitiously. As a core two-qubit operation in quantum computing, the CNOT gate modifies the target qubit state conditionally based on the control qubit, which attackers may leverage to entangle their auxiliary qubits with protocol-critical quantum states for unauthorized information extraction. Security assessments of related protocols [23,36,37,38,39,40] emphasize that rigorous analysis of such attack vectors is essential to guaranteeing protocol-level security.

In our protocol, Bob still acts as the attacker. To obtain the information sent by Alice to Charlie, Bob attempts to use the CNOT attack. First, Bob needs to generate an initial single photon state ${|0\rangle }_{b}$ and perform the CNOT operation, where Charlie’s qubit serves as the control bit and the qubit prepared by Bob serves as the target bit. We use decoy photons to detect eavesdropping behavior. At this stage, the quantum system undergoes unitary evolution into the composite state:

(27) $$\;CNOT{(|0\rangle }_{c}{|0\rangle }_{b}{)=|00\rangle }_{c_{1}b_{1}}\;$$

(28) $$\;CNOT{(|1\rangle }_{c}{|0\rangle }_{b}{)=|11\rangle }_{c_{1}b_{1}}\;$$

(29) $$\;CNOT{(|+\rangle }_{c}{|0\rangle }_{b})=\frac{1}{\sqrt{2}}(|00\rangle +{\left|11\rangle \right)}_{c_{1}b_{1}}\;$$

(30) $$\;CNOT{(|-\rangle }_{c}{|0\rangle }_{b})=\frac{1}{\sqrt{2}}(|00\rangle -{\left|11\rangle \right)}_{c_{1}b_{1}}\;$$

Here, the subscript *c* represents the particle sent from Alice to Charlie. After performing the first CNOT gate operation on the pair {c,b}, the result is $\left\{c_{1},b_{1}\right\}$. Bob then sends $c_{1}$ to Charlie. Charlie applies either a CTRL or SIFT operation to his received particle, transforming $c_{1}$ into $c_{2}$. After completing all operations, Charlie permutes the order of all particles. Consequently, Bob loses the ability to determine the exact positions of the returned particles. This means Bob cannot identify which specific particle requires a CNOT gate operation for each returned particle from Charlie. The detailed scenarios are presented in Table 4.

To facilitate understanding, as shown in Table 4, Alice prepares an initial decoy particle in the state ${|+\rangle }_{c}$. To obtain information, Bob needs to perform a CNOT attack on this particle using the ancillary particle ${|0\rangle }_{b}$. At this point, the system will transform into $\frac{1}{\sqrt{2}}(|00\rangle +{\left|11\rangle \right)}_{c_{1}b_{1}}$. When Charlie selects the CTRL operation and sends the particle back to Alice through the reverse channel, if Bob does not execute the CNOT operation, there is a probability of $\frac{1}{2}$ that Alice will obtain the state |−〉 when measuring $c_{2}$ in the *X* basis. The probability of detection, when the length of such a sequence is *L*, is given by the following formula: if this situation occurs *L* times, then the probability of being detected is $p=1-{\left(\frac{1}{2}\right)}^{L}\left(L\to \infty \right)$. Therefore, Bob must execute the CNOT operation to avoid detection. Since he does not know which particle to perform the CNOT operation on, he has to apply the operation to all the decoy particles. In this case, the state of the target particle $b_{2}$ remains |0〉 all the time, which means that Bob cannot obtain the secret message through the double CNOT attack.

### 4.4. Trojan Horse Attack

Trojan horse attacks typically involve a malicious participant Bob injecting Trojan horse photons into the quantum channel, aiming to analyze the characteristics of reflected or modulated quantum states to determine whether participant Charlie has performed measurement or reflection operations. Specifically, attackers exploit optical imperfections in quantum devices, such as residual polarization crosstalk, phase drifts, or reflection loss variations, combined with high-precision detection schemes to identify deterministic state changes (e.g., polarization rotations or temporal delays) caused by measurements or stochastic noise patterns retained during reflections. However, Charlie can deploy PNS and WF techniques [29,31,41] to detect such eavesdropping attempts prior to executing measurements or reflections, thereby mitigating the attack risk.

## 5. Efficiency and Comparison

According to references [42], the information-theoretic efficiency of the protocol is defined as $\eta =\frac{b_{s}}{q_{t}+b_{t}}$, where $b_{s}$ denotes the total number of shared classical bits, $q_{t}$ represents the quantity of qubits consumed qubits, and $b_{t}$ is the number of classical bits. In this protocol, the length of the secret shared between Alice and the participants is *n*. The lengths of the shared sequences $W_{a}$, $W_{b}$, and $W_{c}$ used for transmitting the secret part are all *n*. Since eavesdropping detection is required, decoy particles of length *l* need to be inserted into both sequences *B* and *C*. Therefore, we calculate that $b_{s}=n$, $q_{t}=3n+2l$, $b_{t}=0$, and the qubit efficiency is $\eta =\frac{n}{3n+2l}$.

Our protocol outperforms analogous schemes in quantum resource efficiency and attack resilience, as systematically validated in Table 5. Unlike Ref. [24], it eliminates entanglement swapping to avoid technical complexity and resource overhead, while supporting specific bit sharing (similar to Refs [18,22]) and uniquely resisting double CNOT attacks. Compared to the W state protocol [21], our design reduces quantum resource consumption via two key optimizations: 1. direct secret-bit encoding into W states to eliminate redundant entanglement generation; 2. strategic decoy photon insertion in only two of three particle streams, minimizing overhead while maintaining eavesdropping detection (Section 4.1). This efficiency edge also distinguishes it from Ref [26]’s architecture, avoiding the heavy quantum bit burden in its sharing process. Such optimizations make our protocol ideal for resource-constrained scenarios where qubit usage must be minimized for practical deployment.

## 6. Conclusions

This paper proposes an efficient and secure SQSS scheme based on *W*-state sharing of specific bits. The scheme enables classical participants to measure in the *Z* basis and collaborate to share secret messages, with no single party able to obtain the secret message alone. We use decoy photons for eavesdropping detection. Security analysis of the protocol demonstrates its strong resistance to eavesdropping, entanglement measurement, double CNOT attacks, and Trojan horse attacks. Subsequently, we compare this protocol with previous ones, highlighting the unique features and advantages of the proposed method.

A key consideration for any secret sharing protocol is its scalability. The proposed three-party protocol can be conceptually extended to accommodate *n* participants through two primary approaches. The most straightforward method is to utilize an *n* partite *W* state, defined as $W_{n}$. In this scheme, the secret is encoded into the choice of the $W_{n}$ state, and each of the *n* participants holds one particle. The reconstruction rule generalizes such that the XOR of all *n* measurement outcomes reveals the secret bit. While this maintains the protocol’s elegant structure, its practical implementation becomes challenging due to the difficulty in generating and distributing large-scale, high-fidelity *n*-particle entangled states. A more practical and robust approach, suitable for near-term implementation, is to structure the protocol hierarchically. A single secret can be shared recursively: for example, Alice first shares the secret with Bob and an intermediate party using the existing three-party protocol. This intermediate party then acts as a new “dealer” to share its share with two other parties, and so on. This tree-like structure builds upon the proven three-party building block, easing the technological requirements at the cost of increased communication rounds and requiring trust in the intermediate parties.

Future research directions include further exploration of these N-party scenarios for flexible scalability with dynamic participant groups, constructing a (T, N)-threshold structured SQSS protocol to enable secret reconstruction by any T participants, and adapting the protocol to real-world applications to ensure effective collaboration among participants with limited quantum capabilities, providing a clear pathway for its practical deployment. 

## Figures and Tables

**Table 1:** Eavesdropping detection of decoy particles.

Initial State	Operation	Alice’s Measure Result
|0〉	CTRL	|0〉
	SIFT	|0〉
|1〉	CTRL	|1〉
	SIFT	|1〉
|+〉	CTRL	|+〉
	SIFT	|0〉 / |1〉
|−〉	CTRL	|−〉
	SIFT	|0〉/ |1〉

$|+\rangle =\frac{1}{\sqrt{2}}(|0\rangle +\left|1\rangle \right)$ and $|-\rangle =\frac{1}{\sqrt{2}}(|0\rangle -\left|1\rangle \right)$.

**Table 2:** Restores specific bit rules.

$m_{i}$	$W_{i}$	$M_{a_{i}}$	$M_{b_{i}}$	$M_{c_{i}}$
0	$W_{0}$	0	0	0
		0	1	1
		1	1	0
1	$W_{1}$	0	0	1
		0	1	0
		1	0	0

**Table 3:** Restores specific bit rules.

Initial State	Bob’s Measurement Basis	Charlie’s Operation	Alice’s Detection Probability
|0〉	*Z*	SIFT	0
		CTRL	0
	*X*	SIFT	50%
		CTRL	50%
|+〉	*Z*	SIFT	0
		CTRL	50%
	*X*	SIFT	0
		CTRL	0

**Table 4:** Bob’s double CNOT attack.

CNOT		Operation	CNOT	
c,b	$c_{1},b_{1}$		$c_{2},b_{1}$	$c_{3},b_{2}$
|0〉|0〉	|00〉	SIFT	|00〉	|0〉|0〉
		CTRL	|00〉	|0〉|0〉
|1〉|0〉	|11〉	SIFT	|11〉	|1〉|0〉
		CTRL	|11〉	|1〉|0〉
|+〉|0〉	$\frac{1}{\sqrt{2}}(|00\rangle +\left|11\rangle \right)$	SIFT	|00〉/|11〉	|0〉|0〉/|10〉
		CTRL	$\frac{1}{\sqrt{2}}(|00\rangle +\left|11\rangle \right)$	|+〉|0〉
|−〉|0〉	$\frac{1}{\sqrt{2}}(|00\rangle -\left|11\rangle \right)$	SIFT	|00〉/|11〉	|0〉|0〉/|10〉
		CTRL	$\frac{1}{\sqrt{2}}(|00\rangle -\left|11\rangle \right)$	|−〉|0〉

**Table 5:** Performance comparison of other SQSS protocols.

Protocol	Ref. [18]	Ref. [21]	Ref. [22]	Ref. [24]	Ref. [25]	Ref. [26]	Our
Quantum resource	Bell state	*W* state	Bell state	cluster state	χ-type state	GHZ-type state	*W* state
Qubit efficiency	$\frac{n}{8n}$	$\frac{n}{24n}$	$\frac{n}{2n+2l}$	$\frac{n}{6n}$	$\frac{n}{40n}$	$\frac{n}{5n}$	$\frac{n}{3n+2l}$
Sharing secret	Specific	Unspecific	Specific	Unspecific	Unspecific	Unspecific	Specific
Resist the CNOT attack	No	No	No	No	No	No	Yes
Resist the trojan horse attack	No	Yes	Yes	Yes	Yes	Yes	Yes
Entanglement swapping	No	No	No	Yes	No	No	No
Transport structure	Distributed	Distributed	Distributed	Distributed	Distributed	Circular	Distributed

## Data Availability

The data presented in this study are available in the article.
